# Comparison of clinical characteristics and mortality risk between patients with cholangiocarcinoma: A retrospective cohort study

**DOI:** 10.3389/fsurg.2022.1037310

**Published:** 2023-02-15

**Authors:** Yaming Liu, Yanhong Wang, Yaqi Yu, Haopeng Luo, Maochuan Zhen, Jianlin Ren

**Affiliations:** ^1^ Department of Gastroenterology and Hepatology, Xiamen University Zhongshan Hospital, Xiamen, China; ^2^ Department of Digestive Diseases, School of Medicine, Xiamen University, Xiamen, China; ^3^ Department of Epidemiology & Biostatistics, School of Basic Medicine Peking Union Medical College & Institute of Basic Medical Sciences Chinese Academy of Medical Sciences, Beijing, China; ^4^ Department of Pathology, Xiamen University Zhongshan Hospital, Xiamen, China; ^5^ Department of Hepatobiliary Surgery, Xiamen University Zhongshan Hospital, Xiamen, China

**Keywords:** cholangiocarcinoma, biliary obstruction, liver function, survival, perihilar cholangiocarcinoma

## Abstract

**Background:**

Cholangiocarcinoma (CCA) is primary liver cancer originating from the biliary tract. The epidemiology of CCA is diverse across the globe. There are no reliably effective options for systemic therapy and CCA outcomes are poor. Herein, we examined the association between overall survival and clinical characteristics of CCA patients in our region.

**Methods:**

We included 62 CCA cases diagnosed between 2015 and 2019. Demographics, clinical history, therapeutic procedures, and concomitant diseases were abstracted. Patient survival was obtained from a household registration system.

**Results:**

The cohort was 69% male and 31% female, with 26 (42%) iCCA, 27 (44%) pCCA, and 9 (15%) dCCA. No age differences were observed between the three subtypes. Bile duct and metabolic disorders were the major concomitant diseases and showed varying associations with CCA subgroups. Serum triglycerides (TG) were higher in pCCA and dCCA than iCCA patients (*p* < 0.05), and TG and total cholesterol (TC) were highest among pCCA patients with cholelithiasis. Liver function appeared significant difference between iCCA, pCCA and dCCA subtypes (*p* < 0.01), and also in the subgroups without cholelithiasis (*p* < 0.01). The obstructive jaundice in pCCA patients was associated with survival time after surgery, and the presence of cholelithiasis was also another influential factor.

**Conclusion:**

We found that pCCA was more frequently associated with metabolic disorders compared to iCCA and dCCA. Postoperative survival was also associated with the degree of jaundice in pCCA compared to iCCA or dCCA. And biliary drainage is an important predictor of outcome of pCCA.

## GRAPHICAL ABSTRACT

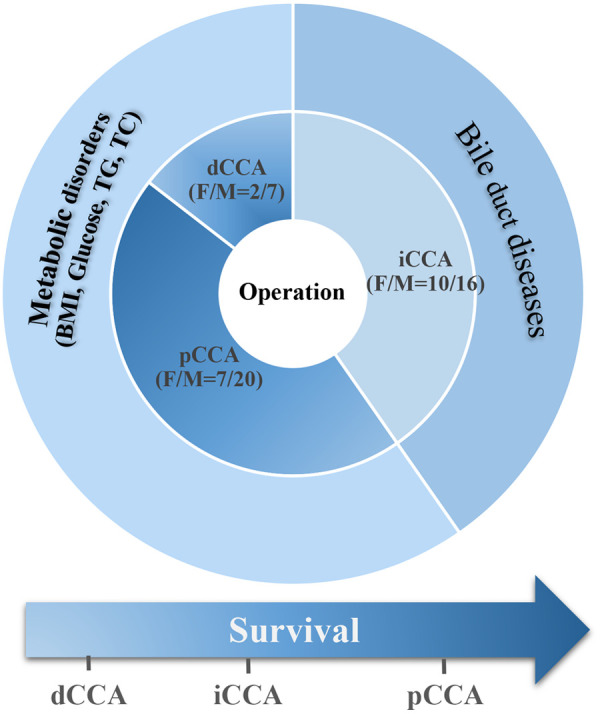

Graphic Abstract. Summary of the survival and clinical characteristics in CCA patients. We have similar numbers of patients diagnosed with iCCA and pCCA (26 and 27 cases, respectively), both of which are significantly higher than those with dCCA (9 cases). There were more male than female patients in our cohort. Bile duct diseases and metabolic disorders were the major concomitant diseases in our CCA patient cohort, showing various association with CCA subgroups. Though all patients underwent operative treatment, the postoperative survival times were still short and there was no significant difference between survival of patients with the different subtypes of CCA.

## Introduction

Cholangiocarcinoma (CCA) are tumors arising from the intrahepatic biliary tract (iCCA) and extrahepatic (eCCA) bile ducts, which is further classified into perihilar (pCCA) and distal (dCCA) on the basis of anatomical location (1). These three subtypes not only differ in localization but also in clinicopathologic characteristics, risk factors and mutational profiles, which can reflect their pathogenesis and are associated with their outcomes (2, 3). Considering the poor prognosis of patients with CCA, improvements in early-stage diagnosis and effective therapeutic options are urgently needed (4, 5).

The complexity of CCA pathogenesis highlights the need for a multidisciplinary, translational, and systemic approach to this malignancy. Surgical resection remains the mainstay of potentially curative treatment for all three disease subtypes, whereas liver transplantation after neoadjuvant chemoradiation is restricted to a subset of patients with early stage pCCA. For patients with advanced-stage or unresectable disease, locoregional and systemic chemotherapeutics are the primary treatment options (6). Improvements in external-beam radiation therapy have facilitated the treatment of CCA. Moreover, advances in comprehensive whole-exome and transcriptome sequencing have defined the genetic landscape of each cholangiocarcinoma subtype (7–9). However, effective treatment for advanced CCA is still a major challenge.

In this study, we retrospectively analyze the clinical characteristics and outcomes of CCA from a single-center from 2015 to 2019.

## Materials and methods

### Study design

**Figure 1:**
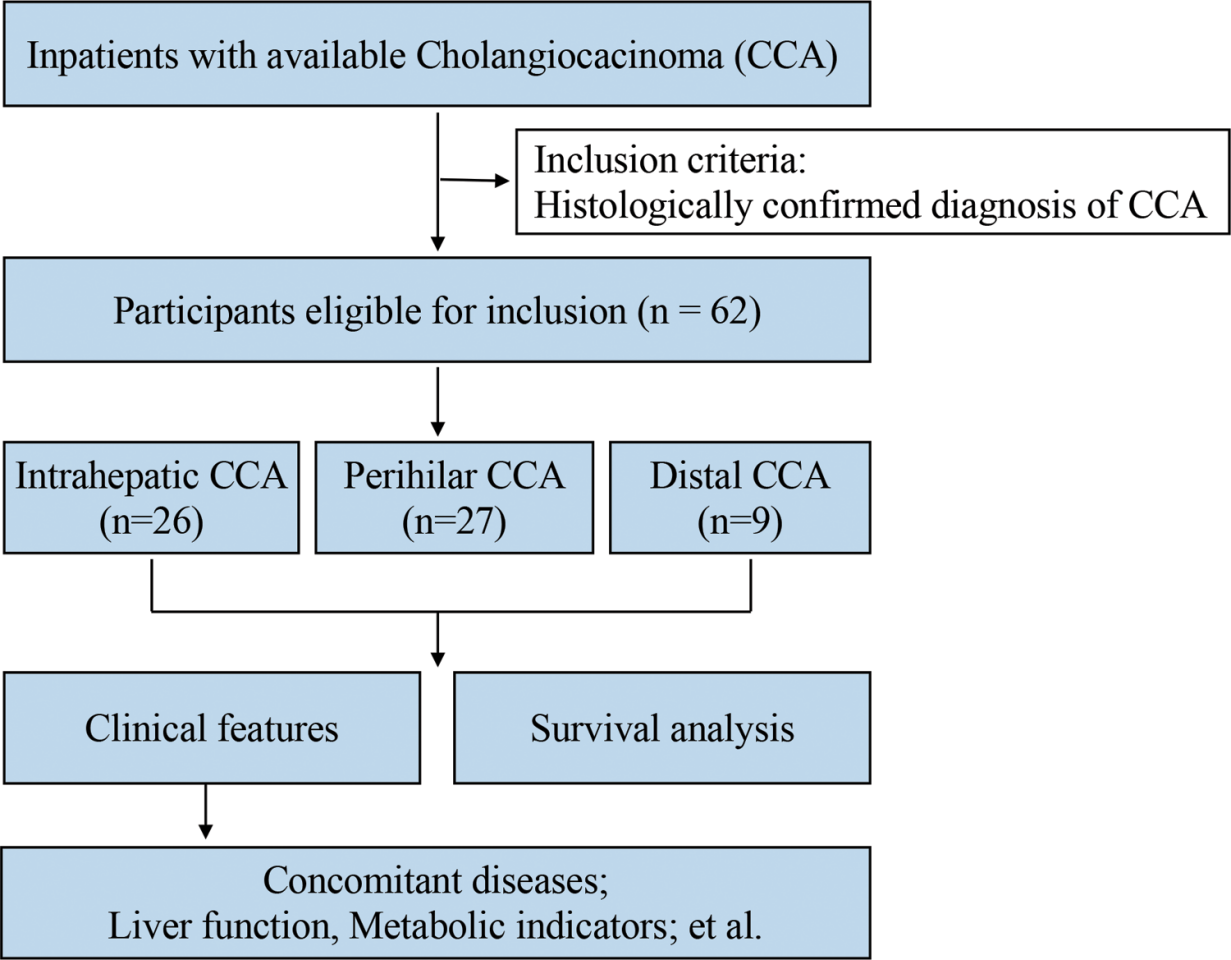
Participant selection.

The present retrospective, single-center study was performed to investigate the clinical features and survival of CCA patients who received operative treatment at Zhongshan Hospital Xiamen University in southeast China between January 1, 2015, and December 31, 2019 (Figure 1). Patient vital status was updated on December 31, 2020. Inclusion criteria were histologically confirmed diagnosis of CCA (all subtypes). At follow-up process, the postoperative patients were healthy, we set the terminate time as 3 years after surgery because postoperative survival time in all these patients was almost not more than 3 years and only two patients' death occurred 4 and 6 years after surgery, respectively. All the other postoperative patients were alive till to December 31, 2020.

This study had obtained ethics approval from the ethics committee at Xiamen University Zhongshan Hospital (xmzsyyky trial no. 2020-109).

### Patient data

Clinical information was abstracted from the medical record system, including patient demographics [age, sex, body mass index (BMI), date of surgery, date of death], serum biochemical indicators (serum lipid levels, liver function, renal function, coagulation function), tumor markers, pathological diagnosis, imaging, therapeutic strategies and concomitant diseases. The major outcome was postoperative survival. The date of death was obtained from the household registration system. Survival analysis covered the time quantum from the time of operation to the date of death. Cutoff date was 3 years after surgery for those healthy patients during follow-up.

### Statistical analysis

Quantitative variables are expressed as medians and ranges and were compared using a nonparametric test. The Chi-square test was used to determine the random distribution of variables. Fisher's exact test was used for the small number of events. Survival curves were calculated according to the Kaplan–Meier method. The Log-rank test was used for survival analysis. *p* values <0.05 was considered statistically significant. All statistical analyses were performed using IBM SPSS Statistics 23.0 software.

## Results

### Patient characteristics

In total, 62 CCA patients were included (Table 1). There were similar numbers of patients diagnosed with iCCA and pCCA, 26 (41.94%) and 27 (43.55%), respectively, both of which are significantly higher than those with dCCA, 9 (14.52%) of cases. The median ages at diagnosis of CCA subtypes were 58, 59, and 58 years, with no age differences observed between the three subtypes. The majority of patients 43 (69.35%) were male.

**Table 1:** Demographics of CCA patients in this study.

	iCCA	pCCA	dCCA	*p* value
Cases (number)	26	27	9	–
Age (years)	58.0 (50.5–64.0)	59.0 (54.0–68.0)	58.0 (49.0–63.0)	0.590
**Sex (F/M)**				0.331
Female (*N*, %)	10 (38.46%)	7 (25.93%)	2 (22.2%)	
Male (*N*, %)	16 (61.54%)	20 (74.07%)	7 (77.78%)	
**Concomitant diseases**				
Cholecystitis	11 (42.31%)	8 (29.36%)	3 (33.33%)	0.589
Gallstone	5 (19.23%)	7 (25.93%)	0	0.285
Hepatolithiasis	8 (30.77%)	2 (7.41%)	1 (11.11%)	0.066
Choledocholithiasis	6 (23.08%)	3 (11.11%)	2 (22.22%)	0.534
Cholangitis (non-PSC)	4 (15.38%)	2 (7.41%)	3 (33.33%)	0.138
HBsAg positive	2 (7.69%)	2 (7.41%)	3 (33.33%)	0.108
HBsAg positive plus cirrhosis	2 (7.69%)	1 (3.70%)	0	0.759
Fatty liver	3 (11.54%)	1 (3.70%)	0	0.370
Hyperlipemia	3 (11.54%)	8 (29.63%)	4 (44.44%)	0.084
Obesity	4 (15.38%)	7 (25.93%)	2 (22.22%)	0.698
Hypertension	4 (15.38%)	8 (29.63%)	1 (11.11%)	0.394
Diabetes mellitus	2 (7.69%)	5 (18.52%)	0	0.342

We found that bile duct diseases, metabolic disorders, chronic liver diseases, especially chronic HBV, and endocrine diseases were the major concomitant diseases in the CCA patient cohort and they showed various associations with CCA subgroups. The bile duct diseases were most common in iCCA patients. Metabolic disorders and bile duct diseases were both common in pCCA patients. Though there were fewer dCCA cases, metabolic disorders and bile duct diseases were still the most common accompanying diseases.

### BMI, glucose and lipid levels in CCA patients

Bile duct diseases were closely associated with disorders of lipid metabolism. To assess the major concomitant diseases of the CCA patients, we analyzed serum metabolic indicators in patients with the subtypes of CCA. Further, we stratified the analyses by the presence of cholelithiasis. The results are shown in Table 2. Serum triglyceride (TG) levels were significantly higher in eCCA patients compared to iCCA patients (*p* < 0.05), and levels of TG and total cholesterol (TC) were the highest among pCCA patients with cholelithiasis. There was no significant difference in BMI and glucose levels between patients with different subtypes of CCA or after further stratification based on cholelithiasis. In summary, hyperlipemia and cholelithiasis were the main concomitant diseases in pCCA patients.

**Table 2:** BMI, glucose and lipid levels in CCA patients.

	iCCA	pCCA	dCCA	*p* value	Non-Cholelithiasis			*p* value	Cholelithiasis			*p* value
					iCCA	pCCA	dCCA		iCCA	pCCA	dCCA	
Body mass index (kg/cm^2^)	21.10 (19.14, 22.38)	21.11 (19.56, 23.47)	22.03 (19.96, 26.22)	0.455	21.63 (19.88, 25.00)	20.96 (19.31, 23.47)	22.03 (19.96, 26.22)	0.442	20.83 (17.51, 22.24)	22.18 (19.56, 23.64)	–	0.285
Glucose (mmol/l)	5.59 (4.92, 5.85)	5.91 (4.94, 7.76)	5.72 (4.73, 7.22)	0.318	5.60 (4.78, 6.06)	5.85 (4.90, 7.80)	5.72 (4.61, 7.01)	0.669	5.31 (5.00, 5.86)	6.65 (5.08, 7.71)	6.31 (5.19, 7.43)	0.278
Triglyceride (mmol/l)	0.95 (0.85, 1.24)	1.57 (1.12, 2.25)	1.53 (0.90, 2.00)	0.011**	4.49 (3.55, 6.23)	4.49 (3.37, 4.96)	4.54 (3.63, 5.00)	0.781	3.95 (3.81, 5.37)	5.42 (4.77, 6.95)	3.92. (3.92, 3.92)	0.036*
Total cholesterol (mmol/l)	3.95 (3.64, 5.36)	4.80 (3.83, 5.55)	4.47 (3.70, 4.95)	0.593	0.94 (0.76, 1.26)	1.36 (1.04, 1.83)	1.71 (0.88, 2.05)	0.177	1.05 (0.89, 1.23)	2.18 (1.18, 3.14)	1.35 (1.35, 1.35)	0.017**

* There is significant difference at triglyceride and total cholesterol levels in the subgroup of pCCA patients with cholelithiasis compared with the other two groups, *p *< 0.05.

** There is significant difference at triglyceride level in iCCA patients compared with pCCA and dCCA groups, *p *< 0.01.

### Liver function in CCA patients

Notably, CCA patients develop obstructive jaundice as the disease progressed and produces obstruction of both right and left hepatic ducts or the common hepatic duct in the case of pCCA or the common bile duct in the case of dCCA. iCCA with extensive intrahepatic metastases and involvement of the peripheral biliary tree can also result in jaundice. Obstructive jaundice severely limits therapeutic options as it limits the ability to deliver systemic therapies, which are typically metabolized and excreted by the liver. In this study, we found the indicators of liver function including alanine aminotransferase (ALT), aspartate aminotransferase (AST), gamma-glutamyl transpeptidase (GGT), alkaline phosphatase (ALP), total bilirubin (TBIL), and direct bilirubin (DBIL) showed significant differences between iCCA, pCCA and dCCA subtypes (*p* < 0.01) (Table 3). Additionally, the biliary obstructive indicators GGT, ALP, TBIL and DBIL were obviously higher in pCCA and dCCA compared with iCCA, and also showed the same tendency in the subgroups without cholelithiasis (*p* < 0.01). But no significant difference was observed between the subgroups of pCCA and dCCA with cholelithiasis (*p* > 0.05). From the above, we found good liver function in iCCA patients was required for them to be eligible for surgical resection, while for eCCA patients, particularly pCCA patients, there was a greater potential for successful surgical resection even with numerically worse liver tests.

**Table 3:** Liver function in CCA patients.

	iCCA	pCCA	dCCA	*p* value	non-Cholelithiasis			*p* value	Cholelithiasis			*p* value
					iCCA	pCCA	dCCA		iCCA	pCCA	dCCA	
ALT	24.40 (16.00, 71.65)	87.85 (43.78, 146.35)	96.90 (33.50, 434.80)	0.002**	21.35 (15.10, 39.85)	107.45 (53.13, 174.63)	96.90 (29.90, 395.80)	0.001**	58.40 (20.30, 236.00)	59.90 (40.25, 118.33)	409.65 (58.13, 573.90)	0.347
AST	29.00 (19.35, 76.45)	78.25 (43.65, 116.33)	64.80 (28.85, 263.45)	0.018*	26.85 (21.43, 42.58)	86.10 (48.30, 119.08)	42.10 (28.20, 229.40)	0.006**	33.00 (18.20, 202.00)	70.65 (43.15, 121.33)	474.90 (48.60, 681.30)	0.509
GGT	111.85 (45.85, 288.00)	365.50 (149.05, 770.78)	600.90 (231.75, 626.30)	0.005**	69.50 (29.70, 320.20)	365.50 (177.83, 725.65)	618.60 (73.00, 215.60)	0.014*	120.60 (89.70, 289.30)	396.50 (120.35, 1054.80)	441.85 (292.88, 387.45)	0.166
ALP	117.90 (86.55, 253.43)	406.00 (245.55, 509.28)	270.40 (179.90, 337.30)	0.000**	98.90 (61.95, 241.95)	393.50 (273.00, 486.68)	290.00 (215.60, 449.30)	0.001**	133.00 (94.60, 318.60)	479.55 (189.75, 612.25)	179.90 (129.45, 157.95)	0.085
ALB	39.12 (35.87, 44.45)	36.74 (33.00, 40.92)	40.57 (36.80, 41.82)	0.149	42.32 (37.67, 47.62)	37.22 (34.20, 42.71)	40.57 (36.70, 41.90)	0.179	38.17 (30.77, 42.35)	33.71 (30.21, 40.00)	39.52 (27.98, 48.85)	0.373
CHE	5759.50 (3779.00, 7712.50)	5154.00 (2973.00, 7257.00)	6707.00 (4093.00, 7027.00)	0.564	6956.00 (4421.50, 8390.25)	5361.00 (2363.00, 7333.50)	6707.00 (4093.00, 7027.00)	0.429	4978.50 (3236.75, 6776.50)	5150.00 (3828.00, 6578.00)	–	1.000
TBIL	13.40 (10.50, 17.60)	174.39 (53.70, 269.20)	80.00 (44.85, 168.50)	0.000**	12.15 (10.48, 16.88)	162.20 (44.50, 245.18)	80.00 (65.20, 218.30)	0.013*	13.60 (10.90, 21.26)	174.39 (89.00, 291.49)	63.70 (18.38, 94.73)	0.043*

**There is obvious difference at ALT, GGT, ALP, TBIL and DBIL levels in iCCA patients compared with pCCA and dCCA groups, *p *< 0.01.

*There is significant difference at AST level in iCCA patients compared with the other two groups, *p *< 0.05.

**There is obvious difference at ALT, AST, ALP and DBIL levels in the subgroup of iCCA patients without cholelithiasis compared with pCCA and dCCA groups, *p *< 0.01.

*There is significant difference at GGT and TBIL levels in the subgroup of pCCA patients without cholelithiasis compared with the other two groups, *p *< 0.05.

*There is significant difference at TBIL level between iCCA, pCCA and dCCA patients with cholelithiasis, *p *< 0.05.

### The pathological characteristics of CCA patients

Then, we summarized the pathological characteristics of these CCA patients (Table 4). Low differentiated adenocarcinoma was the major histological type in iCCA and pCCA subtypes.

**Table 4:** The pathological characteristics of CCA patients.

Subtype of CCA	Cases	Histological Classification			*p*	Tumor Size		*p*	Differentiation			*p*	Vessel tumor embolus		*p*	Lymphatic metastasis		*p*	Perineural invasion (PNI)		*p*	Distant metastasis		*p*
		Adeno-carcinoma	Cystadeno-carcinoma	Other mixed type		<5 cm	>5 cm		Well	Moderate	*P*oorly		With	Without		With	Without		With	Without		With	Without	
iCCA	26	21	1	4	<0.001	8	15	0.210	7	18	1	<0.001	9	14	0.405	5	18	0.011	16	7	0.093	6	17	0.035
pCCA	25	23	0	2	<0.001	20	5	0.004	5	15	4	<0.001	7	15	0.134	7	15	0.134	19	3	0.001	6	19	0.015
dCCA	7	7	0	0	–	5	1	0.219	1	4	2	0.839	1	5	0.219	2	4	0.687	4	2	0.687	5	4	1.000

Small tumor size is one of the characteristics in pCCA subtype. No significant differences were observed between vessel tumor embolus or perineural invasion in all subtypes of CCA. There were less lymphatic metastasis and distant metastasis in iCCA subtype.

### Survival

**Figure 2:**
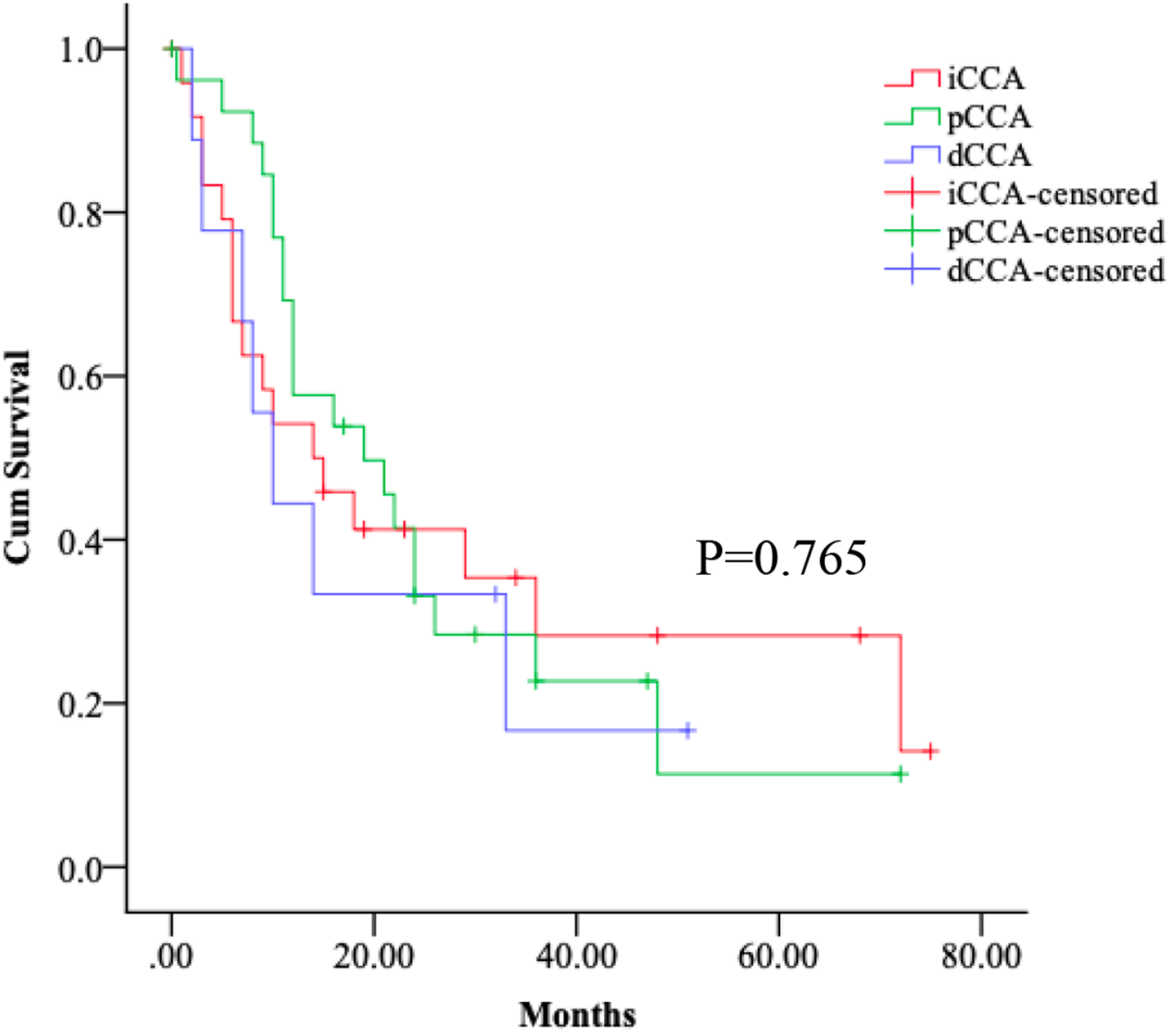
Survival of the subtypes of CCA patients. Kaplan–Meier curves show no differences in the postoperative median follow-up time between iCCA, pCCA and dCCA patients, log-rank *p* > 0.05.

In this study of CCA patients undergoing surgical resection, we found that most had early stage disease at the time of surgery. However, the malignant potential of CCA was high and survival was short despite their eligibility for surgical resection. Thus, it is valuable to understand these postoperative patients' survival. The median overall survival from diagnosis for patients with operative treatment in iCCA, pCCA and dCCA groups were 14 [95% confidence interval (CI), 3.5–24.5] months, 19 (95% CI, 7.1–30.9) months, and 10 (95%CI, 4.2–15.8) months, respectively. We found no differences in the postoperative median follow-up time between iCCA, pCCA and dCCA patients (*p* > 0.05) (Figure 2).

**Figure 3:**
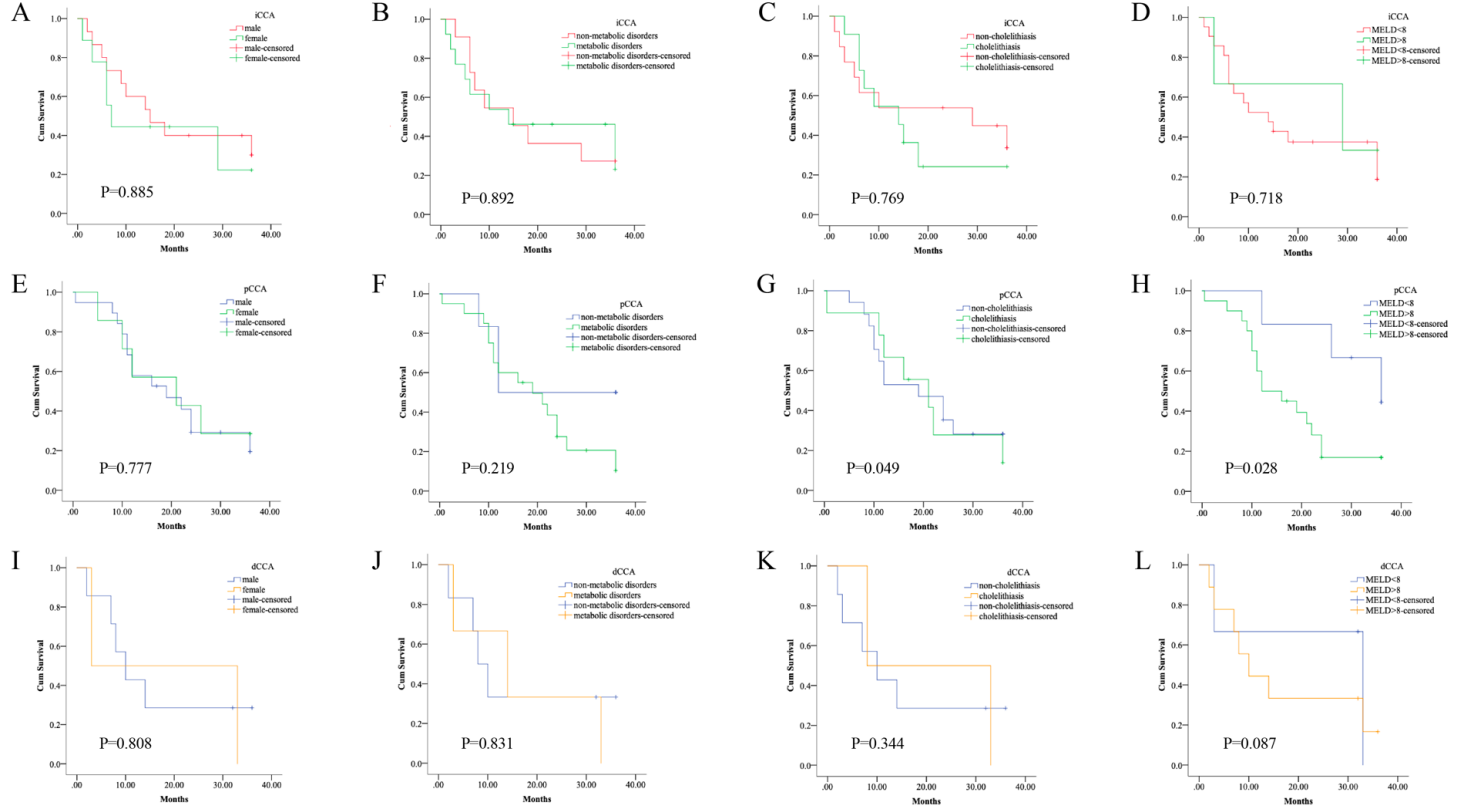
Survival of the subtypes of CCA patients by co-existing conditions. The subtypes of CCA patients were classified into different subgroups by sex, by the degree of jaundice, by the presence of cholelithiasis, or metabolic disorders, respectively. Kaplan–Meier curves show the different survival times. (**A–D**) There was no significant difference in the median survival time of the subgroups of iCCA patients based on different conditions, log-rank *p* > 0.05. (**E–H**) There was a significant difference in the median survival time of pCCA patients with the presence of cholelithiasis, or with different levels of obstructive jaundice, log-rank *p* < 0.05. However, there was no significant difference in the median survival time of the subgroups of iCCA patients based on the other conditions, log-rank *p* > 0.05. (**I–L**) There was no significant difference in the median survival time of the subgroups of dCCA patients based on different conditions, log-rank *p* > 0.05.

Next, the patients were classified into different subgroups with the degree of jaundice base on the total bilirubin level, we found a significant difference in the median survival time of pCCA patients while no such difference was found in iCCA or dCCA patients (Figures 3D,H,E). We also found the presence of cholelithiasis was slightly associated with the postoperative survival of pCCA patients (Figures 3C,G,K). No association was observed between postoperative survival of CCA patients and sex or metabolic disorders (Figures 3A,B,E,F,H,J). In brief, postoperative survival of pCCA was particularly associated with liver function compared to patients with iCCA or dCCA.

## Discussion

Globally, incidence and mortality rates of CCA show substantial geographical variation. Presumably, the variations in incidence reflect, at least partly, differences in geographical risk factors as well as genetic determinants (10, 11). Therefore, it is important to characterize and analyze the features and determinants of outcomes of CCA patients in different geographic regions. The major findings of this retrospective study are that (1) bile duct diseases and metabolic disorders are the key concomitant diseases in the subtypes of CCA patients; (2) the liver function is a critical determinant of early operative treatment for the subtypes of CCA patients; (3) postoperative survival in pCCA patients is particularly associated with their liver function. Figure 4 summarizes these key findings, which are discussed in greater detail below.

First, of note, over the past few decades, the incidence rates of CCA appear to be changing and the subtypes of CCA also show distinct epidemiological trends (12–14). In this study, we found that bile duct diseases and metabolic disorders are the main accompanying diseases for all subtypes of CCA patients. Additionally, hyperlipidemia and concomitant cholelithiasis are key accompanying factors in pCCA risk in our region. It has been well documented that metabolic reprogramming is a hallmark of cancer, and highly proliferative CCA cells are strongly lipid-dependent. Recently, targeting metabolism has been proposed for cancer therapy (15, 16). Above all, we should follow and focus on the patients with bile duct diseases accompanying metabolic disorders.

Next, liver function is a critical determinant of eligibility for early operative treatment for patients with all subtypes of CCA. With CCA disease progression, the liver tests and function gradually worsen and patients fairly rapidly develop unresectable disease. Patients with early stage CCA are potential candidates for surgical resection. Patients with advanced unresectable CCA have no reliably effective therapeutic options and poor survival (17–19). In this study, all CCA patients underwent operative treatment. And biliary obstructive indicators showed obvious differences between the iCCA, dCCA and pCCA subtypes. For iCCA patients, GGT, ALP, TBIL and DBIL levels predominantly affect the decision of surgery, particularly those with underlying liver disease. For eCCA patients, since obstruction of larger bile ducts, they are more likely to safely tolerate operative treatment. Thus, liver function is the key factor to decide the safe of surgery.

We addressed the potential role of cholelithiasis in outcomes of CCA patients. When the entire patient cohort was divided into two subgroups based on cholelithiasis, the same differences in factors determining eligibility for surgical resection were observed whether there was concomitant cholelithiasis or not. Thus, iCCA patients with earlier stage CCA tumors who show declines in liver function are ineligible for operative treatment regardless of the presence of cholelithiasis. In regard to eCCA patients, cholelithiasis is a potential factor affecting the liver function of patients.

Overall, our results confirm that the malignant aggressiveness of CCAs is high, with a high rate of recurrence after surgery with curative intent and a relatively poor prognosis compared to other cancer types. Although all our patients underwent operative treatment, the postoperative survival time was still short and the median overall survival durations were less than one a half year. Our study shows that postoperative survival of pCCA patients is particularly associated with their preoperative liver function. Therefore, further studies should be carried out for increasing the feasibility of surgical resection and reducing the likelihood of post-operative recurrence.

In summary, we found that CCA patients were more likely to have metabolic disorders, and that liver function is a critical determinant in the early operation treatment and survival of pCCA patients. And these findings provide a framework for further prospective studies on diagnosis, treatment and prognosis for the subtypes of CCA. However, further and larger prospective studies should improve our understanding of the determinants of the incidences of CCA subtypes in our region.

## Data Availability

The original contributions presented in the study are included in the article/Supplementary Material, further inquiries can be directed to the corresponding author/s.
